# Antigenic Cartography Indicates That the Omicron BA.1 and BA.4/BA.5 Variants Remain Antigenically Distant to Ancestral SARS-CoV-2 after Sputnik V Vaccination Followed by Homologous (Sputnik V) or Heterologous (Comirnaty) Revaccination

**DOI:** 10.3390/ijms241310493

**Published:** 2023-06-22

**Authors:** Ekaterina A. Astakhova, Alexey A. Morozov, Maria G. Byazrova, Maria M. Sukhova, Artem A. Mikhailov, Aygul R. Minnegalieva, Andrey A. Gorchakov, Alexander V. Filatov

**Affiliations:** 1 Laboratory of Immunochemistry, National Research Center Institute of Immunology, Federal Medical Biological Agency of Russia, 115522 Moscow, Russia; 2 Department of Immunology, Faculty of Biology, Lomonosov Moscow State University, 119234 Moscow, Russia; 3 Ministry of Science and Higher Education of Russia, RUDN University, 117198 Moscow, Russia; 4 Laboratory of Synthetic and Evolutionary Biology, Okinawa Institute of Science and Technology, Okinawa 904-0495, Japan; 5 Laboratory of Immunogenetics, Institute of Molecular and Cellular Biology, Siberian Branch of the Russian Academy of Sciences, 630090 Novosibirsk, Russia

**Keywords:** SARS-CoV-2, COVID-19, revaccination, virus neutralization, antigenic cartography

## Abstract

The rapid emergence of evasive SARS-CoV-2 variants is an ongoing challenge for COVID-19 vaccinology. Traditional virus neutralization tests provide detailed datasets of neutralization titers against the viral variants. Such datasets are difficult to interpret and do not immediately inform of the sufficiency of the breadth of the antibody response. Some of these issues could be tackled using the antigenic cartography approach. In this study, we created antigenic maps using neutralization titers of sera from donors who received the Sputnik V booster vaccine after primary Sputnik V vaccination and compared them with the antigenic maps based on serum neutralization titers of Comirnaty-boosted donors. A traditional analysis of neutralization titers against the WT (wild-type), Alpha, Beta, Delta, Omicron BA.1, and BA.4/BA.5 variants showed a significant booster humoral response after both homologous (Sputnik V) and heterologous (Comirnaty) revaccinations against all of the studied viral variants. However, despite this, a more in-depth analysis using antigenic cartography revealed that Omicron variants remain antigenically distant from the WT, which is indicative of the formation of insufficient levels of cross-neutralizing antibodies. The implications of these findings may be significant when developing a new vaccine regimen.

## 1. Introduction

The rapid evolution of SARS-CoV-2 has resulted in a range of antigenic variants. The currently dominant Omicron lineage has over 30 substitutions in its spike (S) protein and features an increased transmissibility allowing the virus to evade neutralizing antibody responses. As a consequence, vaccines based on the original strain exhibit waning effectiveness against the emerging viral variants, which poses a significant challenge in COVID-19 vaccinology.

To date, over 50 COVID-19 vaccines have been approved (https://covid19.trackvaccines.org/, accessed on 15 May 2023). Most of them are based on the spike sequences derived from the original, ancestral SARS-CoV-2 strain. Some vaccines have been quickly adapted for producing a spike protein from the Omicron subvariants, such as BNT162b2 Bivalent, and mRNA-1273.214 and -222. However, developing and approving these vaccines for widespread use is slower than the emergence of new viral variants. Evaluating virus-neutralizing antibody (NAb) responses is crucial for determining the current vaccines’ efficacy against the immune-evasive strains of the virus.

Various assays have been developed for determining virus neutralization titers (VNTs) of serum samples in laboratories [1,2]. These assays provide extensive but rather fragmented data that are difficult to visualize and interpret when considering multiple variants of concern (VOCs). A more in-depth analysis also requires quantifying the breadth of the antibody response (recognizing several antigenically distinct mutant variants). To address these issues, the method of antigenic cartography has proven itself well. This approach is based on multidimensional scaling [3]. It simplifies neutralization data visualization, and quantifies the antigenic distances (distances between the variants on the antigenic map) and breadth of virus neutralization. Antigenic maps show the viral variants’ clusterization based on how they are recognized by the tested sera. In an advanced version, antigenic cartography makes it possible to reconstruct antibody landscapes and breadth gain plots that in turn allow for quantifying the breadth of neutralization. This parameter is very often qualitatively discussed in the context of various vaccination regimens, but it is cartography that gives this term a clear definition and quantitative assessment. Antigenic landscapes are also used to predict neutralization titers against variants that were not used in making the landscape [4].

Antigenic maps were created for SARS-CoV-2 using sera from donors who had recovered from COVID-19 with the PCR-confirmed SARS-CoV-2 variant or who had been vaccinated against COVID-19 as well as from animals immunized with known viral variants [5,6,7,8,9,10,11,12,13,14,15]. The relatively close positions of the early pandemic variants to each other were shown while Omicron lineage SARS-CoV-2 was far away from them. In particular, specific mutations in the Omicron spike protein have been shown to result in major antigenic differences [7]. This method is particularly useful for monitoring the antibody maturation process after multiple vaccine doses [8,9,10]. Shortening of antigenic distances between ancestral and mutant variants after antigen re-exposure indicates the emergence of cross-reactive serum antibodies [9]. So far, limited data are available for adenovirus-based COVID-19 vaccines [6,15] and none are available for other platforms. 

In the present study, we compared antigenic maps before and after homologous Sputnik V revaccination (Sputnik Light, which uses the first Ad26-based dose of a two-component GAM-COVID-Vac), including the individuals with hybrid immunity, and after heterologous Sputnik V/Comirnaty (BNT162b2, BioNTech/Pfizer) revaccination.

## 2. Results

### 2.1. Study Design

**Figure 1:**
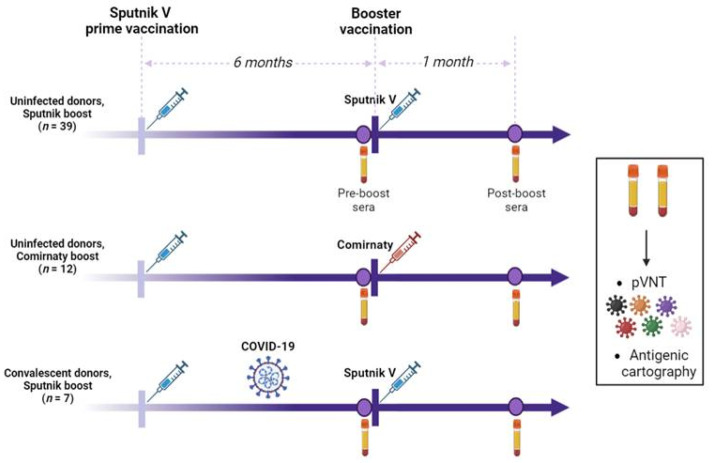
Study design.

Our study included 58 individuals who were primed with two doses of Sputnik V vaccine in 2021 (Figure 1). After six months, 46 individuals received Sputnik V as a booster vaccine, while 12 received Comirnaty. The donors who were recruited to the homologous arm of the study only received the first dose of Sputnik V (Ad26-based) as a booster. In this subgroup, 39 individuals did not have COVID-19 at any time throughout the study (negative for Nucleocapsid-specific antibodies, subgroup “Uninfected donors, Sputnik V booster”), while 7 individuals had hybrid immunity due to a prior infection (subgroup “Convalescent donors, Sputnik V booster”). The “Convalescent” subgroup was determined based on three parameters: clinical data (positive SARS-CoV-2 PCR test), the levels of anti-Nucleocapsid IgG antibodies (more than 5 of a maximum of 15 relative units), and the level of anti-S IgG antibodies (more than 10 of a maximum of 15 relative units) before revaccination (Table 1). To be included in the “Convalescent” subgroup, at least two of the three parameters had to be met.

The study measured the neutralizing antibody titers in sera collected immediately before and one month after the booster using a panel of six pseudoviruses: WT (wild-type), Alpha, Beta, Delta, Omicron BA.1, and BA.4/BA.5.

### 2.2. The Level of Post-Boost SARS-CoV-2 Neutralizing Antibodies (NAbs) Depends on the Type of Booster Vaccine and the History of Exposure to SARS-CoV-2

**Figure 2:**
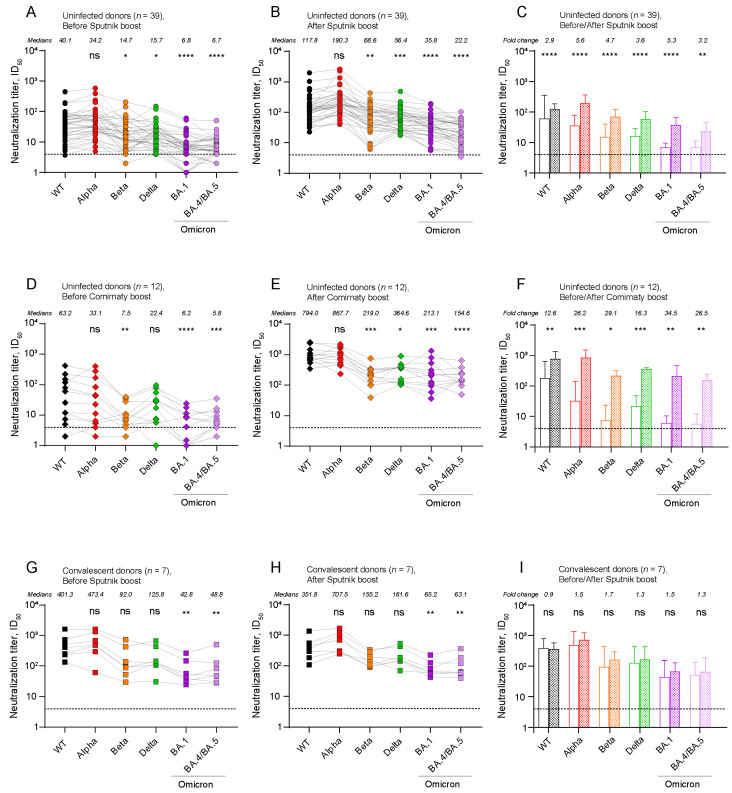
Neutralization antibody titers (ID_50_ values) against SARS-CoV-2 variant pseudoviruses for pre- and post-boost sera from individuals primarily vaccinated with Sputnik V. Sera from the uninfected donors revaccinated with (**A**–**C**) Sputnik V or (**D**–**F**) Comirnaty. Sera from (**G**–**I**) convalescent donors revaccinated with Sputnik V. Asterisks indicate a significant difference when comparing ID_50_ values against the WT with variants. Connecting lines indicate serum from the same individual. (**C**,**F**,**I**) Summary of virus-neutralizing activities of serum samples against the WT and variants before (open bars) and after (solid bars) Sputnik/Comirnaty boost. Asterisks indicate a significant difference when comparing ID_50_ values before and after a boost for each variant. Colors of points and bars correspond to the variants: black–WT, red–Alpha, orange–Beta, green–Delta, violet–Omicron BA.1, pink–Omicron BA.4/BA.5. Medians ± IQR of ID_50_ values are shown. Statistical analysis was performed using the Friedman test and Dunn’s post hoc test, * *p* < 0.05, ** *p* < 0.01, *** *p* < 0.001, **** *p* < 0.0001, ns = not significant.

Pre-boost levels of NAbs against the WT, Alpha, Beta, Delta, Omicron BA.1, BA.4/BA.5 variants were above the baseline in 97%, 100%, 97%, 100%, 82%, 87% of uninfected donors (Sputnik V booster), respectively (Figure 2A). Therefore, the majority of the Sputnik V-vaccinated donors maintain high levels of NAbs for 6 months after the primary vaccination. Only one sample at the post-boost time point remained on the baseline for the neutralization of BA.4/BA.5, while the rest of the samples showed the titers significantly above the baseline (Figure 2B).

We observed that pre-boost neutralization titers against the Beta, Delta, and Omicron variants were significantly lower than those against the WT (*p* < 0.05 for Beta and Delta, *p* < 0.0001 for Omicron variants) (Figure 2A). The activity of the NAbs present in the post-boost sera against the Beta, Delta, and Omicron variants followed the same trend as in the pre-boost samples (*p* < 0.01, *p* < 0.001, *p* < 0.0001 for the WT vs. the Beta, Delta, and Omicron variants, respectively) (Figure 2B).

Next, we examined the serum samples from uninfected donors who received a heterologous Comirnaty booster (Figure 2D,E). When tested against the WT, Alpha, Beta, and Delta variants, 92% of the pre-boost serum samples displayed VNT values above the baseline, whereas only 58% and 83% of samples exceeded the baseline in assays against the BA.1 and BA.4/BA.5 variants, respectively (Figure 2D). After the Comirnaty boost, all VNT values were above the baseline (Figure 2E).

Both the Beta and Omicron variants demonstrated significantly lower neutralization than the WT (*p* < 0.01, *p* < 0.0001, *p* < 0.001 for the Beta, BA.1, and BA.4/BA.5 variants, respectively) in pre-boost sera (Figure 2D). Following the heterologous booster, serum antibodies had lower neutralizing activity against Beta and Omicron variants, and also in contrast to pre-boost sera against the Delta variant compared to the WT (*p* < 0.05, *p* < 0.001, *p* < 0.0001 for Delta, Beta and BA.1, BA.4/BA.5, respectively) (Figure 2E).

The impact of the booster on the convalescent donors was distinctly different from that on the uninfected donors (Figure 2G–I). First of all, the level of pre-boost NAbs in this subgroup was higher than in the uninfected subgroup, and 100% samples exceeded the baseline of virus neutralization across all of the studied variants (Figure 2G). The significant difference in neutralization levels compared to the WT was only observed for the Omicron variants in both pre- and post-boost sera (*p* < 0.01 for both time points and both BA.1 and BA.4/BA.5) (Figure 2G,H).

Finally, we compared the pre- and post-boost level of NAbs with each other for each variant (Figure 2C,F,I). The Sputnik V booster significantly increased the neutralization titers to all of the studied variants in the uninfected subgroup (Figure 2C). We observed the largest increase ID_50_ for the Alpha variant (5.6-fold change, *p* < 0.0001), and the lowest increase for the WT (2.9-fold change, *p* < 0.0001) variant. The titers against the Beta, Delta, BA.1, and BA.4/BA.5 variants increased by 4.7, 3.6, 5.3, and 3.2 times, respectively (*p* < 0.01 for BA.4/BA.5 and *p* < 0.0001 for others). These results suggest that Sputnik V can boost the neutralizing antibody levels in uninfected donors against various variants of SARS-CoV-2, including Omicron.

The Comirnaty booster induced a strong increase in the VNT values against all of the analyzed variants (Figure 2F). The fold changes were higher than those observed with the homologous booster, with increases of 12.6, 26.2, 29.1, 16.3, 34.5, and 26.5 times for the WT, Alpha, Beta, Delta, BA.1, and BA.4/BA.5 variants, respectively.

No changes in VNTs were observed among the recovered donors who received the Sputnik V booster (Figure 2I), thereby suggesting that it was the infection rather than boosting that contributed the most to the neutralization profiles observed.

### 2.3. Antigenic Maps Reveal the Emergence of Booster-Induced Cross-Neutralizing Antibodies

**Figure 3:**
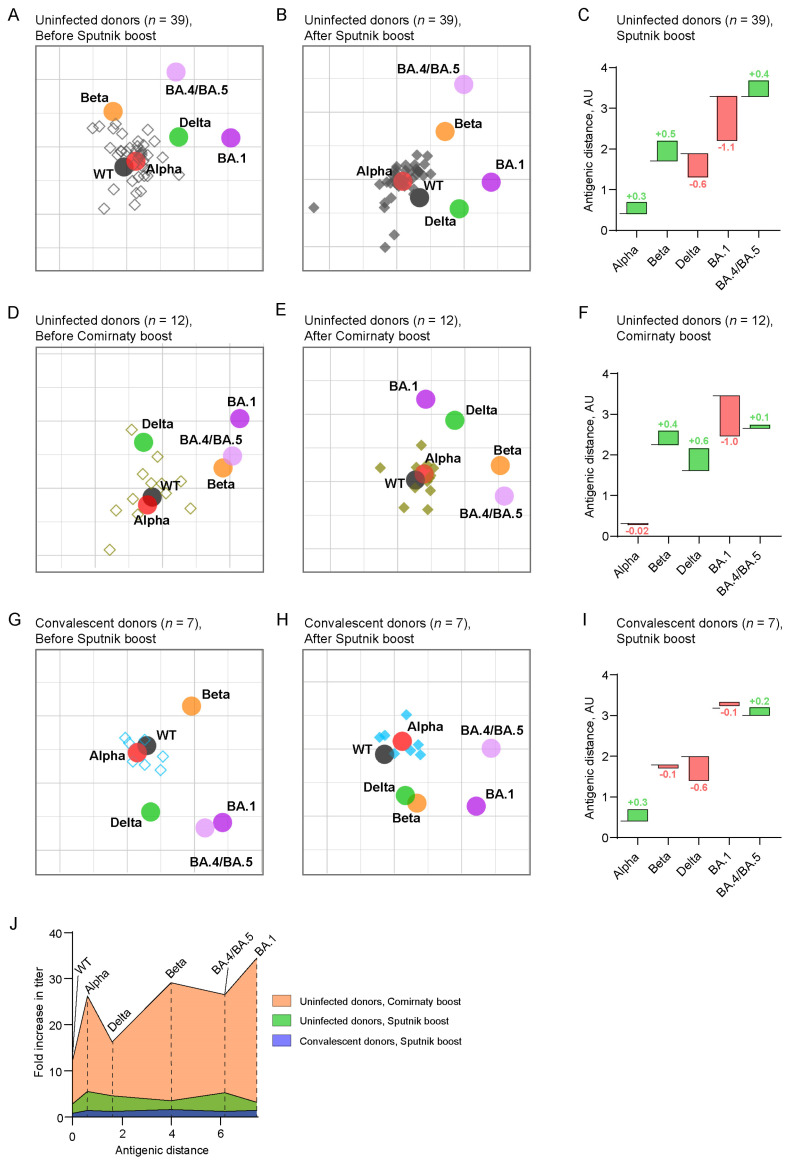
Antigenic cartography of pre- and post-boost serum samples against the wild-type (WT), Alpha, Beta, Delta, and Omicron BA.1 and BA.4/BA.5 variants. The serum samples are shown in open ((**A**,**D**,**G**), before boost) or solid ((**B**,**E**,**H**), after boost) diamonds as follows: uninfected donors with Sputnik V boost (black), uninfected donors with Comirnaty boost (olive), and convalescent donors with Sputnik V boost (blue). Each grid square (one antigenic unit) corresponds to a twofold dilution in the neutralization assay. Antigens are shown as circles and labeled. Hat graphs represent changes in antigenic distances between the WT and spike variants during revaccination (**C**,**F**,**I**). The brim of the hat corresponds to the antigenic distance for pre-boost samples. The hat height indicates the change in the antigenic distance between the pre- and post-boost samples. The green/red hats indicate an increase/decrease in the antigenic distance. The numbers indicate the corresponding changes in the antigenic units. Breadth gain plots of serum samples from individuals primarily vaccinated with Sputnik V and who received homologous with Sputnik V or heterologous with Comirnaty revaccination (**J**). The x-axis represents the antigenic distances of Alpha, Beta, Delta, Omicron BA.1, and BA.4/BA.5 variants from the WT, which were taken from [7,10]. The y-axis represents the fold increase in neutralization titer after Sputnik V or Comirnaty revaccination.

Antigenic maps were created to visualize the relationships between the sera and the VNTs of SARS-CoV-2 spike-pseudotyped lentiviral particles (Figure 3). The positioning of each serum sample on the maps is indicative of its efficacy in neutralizing a particular variant. One grid line on the antigenic maps corresponds to a twofold dilution in the virus neutralization assay (one antigenic unit, AU). We used this scale to examine the changes in the antigenic distances between the WT and the other studied variants to reveal the cross-neutralization activity of the serum samples.

In the subgroup of uninfected donors before Sputnik V revaccination, the serum samples clustered near the WT- and Alpha-based spikes and farther from the Beta variant (Figure 3A). Sputnik V booster led to tighter clustering near the Alpha variant, which corresponds to the maximum increase in ID_50_ against the Alpha variant, and a smaller increase near the WT spike (Figure 3B).

Sera from the “Comirnaty booster” subgroup before revaccination likewise clustered around the WT and Alpha variants. Two sera were close to the Delta variant (Figure 3D), which could be due to an asymptomatic or undetected infection with the Delta variant that disseminated in Russia in 2021 [16]. To control for potential errors related to these two sera, we constructed additional maps where the data from these two donors were excluded (Figure S1). These maps did not show significant differences of antigenic distances between the WT and the variants, and therefore our next analysis was based on the full subgroup (*n* = 12). No sera were observed to cluster near the Beta variant, which was in contrast to the “Sputnik V booster” subgroup. Post-boost sera, again, clustered close to the WT and the Alpha variant (Figure 3E).

In the subgroup of the convalescent individuals, both before and after revaccination, sera clustered close to the WT and the Alpha variant (Figure 3G,H).

Initially, in the samples collected before revaccination, the vaccine-resistant variants BA.1 and BA.4/BA.5 were positioned the farthest from the WT across all three study subgroups (Figure 3A,D,G), which is consistent with previous studies [5,7,15]. The hat graphs showed that the BA.1 after Sputnik V became 1.1 AU closer to the WT in the uninfected donors subgroup, unlike the BA.4/BA.5 variant that became more distant by 0.4 AU (Figure 3C). The Beta, Delta, BA.1, and BA.4/BA.5 variants became approximately equidistant to the WT after Comirnaty revaccination, and the BA.1 variant became closer to WT (1.0 AU) (Figure 3F). The change in antigenic distance between the WT and the BA.4/BA.5 variants was not significant in this subgroup. In the convalescent subgroup, the BA.1 and BA.4/BA.5 variants did not appreciably move relatively to the WT (Figure 3I).

Next, our analysis uncovered the opposite trends in the antigenic distance shifts between the WT and Delta spike variants, depending on the nature of the booster. Specifically, the Sputnik V booster led to a decrease of 0.6 AU in the uninfected and convalescent subgroups. In contrast, after the Comirnaty booster, the antigenic distance increased by 0.6 AU.

Finally, we created a breadth gain plot for all three study subgroups (Figure 3J). Here, the x-axis shows the antigenic distance between the WT and the variants, which were taken from [7,10]. The y-axis corresponds to the boost-induced fold change in VNTs. The area under the curve in the Comirnaty booster subgroup is visually larger than for the other two subgroups. The gain in titer was smaller for the Delta and BA.4/BA.5 variants compared to the earlier viral variants (Alpha and Beta, respectively) than what has been reported previously [10]. That study demonstrated that the fold increase in VNT values corresponds to larger antigenic distance between the vaccine strain (WT) and the variant. The uninfected subgroup deviated from this trend because of smaller fold changes for the Delta, Beta, and BA.1 variants compared to the variants that emerged earlier. The graph for the convalescent donors was essentially parallel and very close to the x-axis.

## 3. Discussion

The most common method for assessing humoral anti-viral immunity induced by vaccination or booster vaccination is through the measurements of VNTs. In the early days of SARS-CoV-2 research, when the ancestral strain or a small number of early viral lineages dominated, the traditional method of evaluating virus neutralizing activity was appropriate. However, as SARS-CoV-2 evolved rapidly, the large amount of neutralization data against multiple variants has made the traditional graphs difficult to interpret.

Under these circumstances, the approach referred to as antigenic cartography provided a more comprehensive understanding of the antigenic diversity of the viral spikes. This approach was initially developed for human seasonal influenza virus but more recently it has also been successfully applied to lyssaviruses, flaviviruses, and other viruses [17,18,19]. Antigenic cartography is now also being used to analyze the antigenic relationships between the SARS-CoV-2 variants [7].

The crucial task of serological studies is to figure out the effectiveness of vaccine-induced humoral responses against a broad range of viral antigens, including those of the emerging and immune-evading viral variants. However, although the term “breadth of virus neutralization” applied to the serum response is widespread in the scientific literature, there is no quantitative assessment for this key parameter. In the case of influenza infections, antigenic cartography is employed to estimate the antigenic distances between the variants and to inform decisions about vaccine strain updates. It is recommended to update the vaccine strain when the antigenic distances between the previous vaccine strain and the new VOCs exceed approximately 2 AU. This recommendation is based on the immunological understanding that the breadth of virus neutralization in the humoral response is insufficient for protection against new variants. Conventionally, the standard reference set of ferret or human sera following primary infection is used to create antigenic maps [7,8]. Because of the fact that SARS-CoV-2-specific human immunity is currently a composite of the previous infection and/or vaccination history, SARS-CoV-2 antigenic maps are now also being constructed using vaccine-elicited sera.

In this study, to quantify and interpret the antigenic relationships between SARS-CoV-2 variants after a booster, we used both the traditional method for assessing the virus-neutralizing activity of sera and antigenic cartography. Some peculiarities of the immune response were revealed via a traditional approach (Figure 2). Both homologous and heterologous regimens of revaccination successfully boosted the antibody response in uninfected donors against all of the studied variants. Despite the fact that both vaccines (Sputnik V and Comirnaty) were based on the ancestral strain, we did not observe the maximum increase in titers against the WT spike-pseudotyped lentiviral particles. This is in agreement with the theory of saturation of high-affinity antibodies [6,20], such as in our example against the WT, and biased selection toward low-affinity antibodies that can neutralize virus variants. In uninfected individuals, VNTs against the Beta and Omicron variants, but not the Alpha variant, were significantly lower than those against the WT both before and after revaccination. A heterologous booster elicited a more robust immune response, which is consistent with the previously published data on a booster with Comirnaty after prime vaccination with ChAdOx1-nCoV-19 [21,22,23].

The Sputnik V booster did not result in increased antibody levels in a subgroup of individuals who had recovered from COVID-19 1–4 months prior to a booster. Previously, for Sputnik V revaccination, it was shown that the magnitude of a boost-induced increase in the antibody levels depended on the initial level of antibodies at the time of revaccination [24]. This effect may have been related to the presence of high levels of specific anti-S antibodies and T-cells, which were sufficient to neutralize the antigen induced by the booster. It is also possible that longer intervals between recovery from infection and subsequent vaccination might be needed to achieve improvements in the antibody levels and the better cross-neutralization of viral variants [25].

Constructing the antigenic maps allowed us to uncover the subtle features of the homologous and heterologous boosters. Despite the fact that both boosters were based on the ancestral spike sequence, a decreased antigenic distance was observed between some of the viral variants, which is probably best explained by the emergence of cross-reactivity in the sera.

Our results also indicate that antigenic distances between the WT and the Delta variant shifted in opposite directions following the different booster vaccination regimens. Therefore, our data agree with the previously published studies demonstrating that a booster with mRNA COVID-19 vaccines leads to the increased distance between the WT and the Delta variant [9,10]. Here, we demonstrated that the homologous adenovirus-based vaccine booster reduces the antigenic distance between the WT and the Delta variant for both uninfected and convalescent individuals. The exact mechanism behind this difference remains to be discovered.

The most significant changes between the WT and the studied variants were observed for BA.1 in uninfected donors for both regimens. In contrast to the Delta variant, these changes were unidirectional, i.e., the distances decreased by nearly 1 AU. Previous research conducted on mRNA vaccines demonstrated that a third booster reduces the antigenic distance between the WT and the BA.1 variant by approximately 2.6 AU [9,10]. Our findings highlight that a single Comirnaty booster, following a priming dose of adenovirus-based vaccine, also led to shorter antigenic distances between the WT and the BA.1 variant but to a lesser extent. 

We only observed a minor increase following a Comirnaty booster, which contrasts with a previous study, where the antigenic distance between the WT and the BA.4/BA.5 variants decreased by nearly 1 AU following the third Comirnaty booster [10]. After the Sputnik V booster, the antigenic distances between the WT and the BA.4/BA.5 variants in our study increased by 0.4 AU. As a result, after the homologous booster, sera were less effective in recognizing the BA.4/BA.5 variants compared to the BA.1 variant. However, following the heterologous booster, both Omicron variants became nearly antigenically equivalent.

To explain the mechanisms responsible for the observed differences following boosting regimens, a more detailed examination of the antigen (S-protein) epitope characteristics induced by the vaccine is required. We hypothesize that the different conformation of the S-protein on the target cells may influence the recognition patterns during antibody maturation. mRNA-based vaccines encode the prefusion-stabilized conformation of the spike protein with two proline substitutions, while Gam-COVID-Vac encodes an unmodified full-length spike [26,27,28].

Our study has several limitations. First, the number of study participants in the convalescent and Comirnaty-revaccinated subgroups was relatively small. Therefore, antigenic maps and the calculated antigenic distances between the WT and variants for these two subgroups are preliminary and need to be confirmed in larger datasets. In contrast, antigenic maps for the uninfected Sputnik boost subgroup are well powered. A second limitation is that some of the donors could have had asymptomatic COVID-19 that remained undetected.

## 4. Materials and Methods

### 4.1. Ethics

Before carrying out any study procedures, written informed consent was given by all of the study participants. The Institute of Immunology’s Medical Ethical Committee assessed and approved the study protocol (#12-1, 29 December 2020).

### 4.2. Volunteers and Samples Collection

A cohort of 59 volunteers was enrolled at the National Research Center Institute of Immunology of The Federal Medical Biological Agency of Russia. Between January and March 2021, all of the subjects received two doses of Gam-COVID-Vac vaccine 21 days apart. Approximately 6 months after the prime, the booster vaccine dose was given. We divided the study cohort into three subgroups: (1) uninfected donors, who received Sputnik Light as a booster (*n* = 39), (2) uninfected donors, who received Pfizer/BioNTech’s as a booster (*n* = 12), and (3) donors who recovered from COVID-19 and subsequently received Sputnik Light as a booster (*n* = 7); Sputnik Light representing the first dose of Gam-COVID-Vac (Sputnik V). As uninfected donors did not have a history of COVID-19, anti-N antibodies were absent from their serum samples before the revaccination. Convalescent donors were infected by SARS-CoV-2 in the period between prime and booster vaccination (Table 1).

Serum samples were collected one day before the booster vaccination and about one month after. Sera were aliquoted and frozen at −70 °C for ELISA and neutralization tests.

### 4.3. SARS-CoV-2 Pseudoviral Particles Production

Three plasmids were utilized to generate lentiviral particles pseudotyped with the SARS-CoV-2 spike: HIV-1 packaging pCMVΔ8.2R (Addgene), transfer pUCHR-GFP (Addgene), and envelope pCAGGS-Swt- Δ1932 [29]. Several plasmid encoding variants of the SARS-CoV-2 spike were obtained via site-specific mutagenesis or gene synthesis (Genewiz) using the pCAGGS-Swt-Δ19 plasmid as a template. This plasmid encoded an ancestral Wuhan-Hu-1 spike (Δ19) that lacked 19 C-terminal amino acid residues and was codon-optimized.

Specifically, these spike variants had the following substitutions: Δ69–70, Δ144, N501Y, A570D, D614G, P681H, T716I, S982A, D1118H (Alpha); L18F, D80A, D215G, Δ241–243, R246I, K417N, E484K, N501Y, D614G, A701V (Beta); T19R, G142D, Δ156–157, R158G, L452R, T478K, D614G, P681R, D950N (Delta); A67V, Δ69–70, T95I, G142D, Δ143–145, Δ211, L212I, ins214EPE, G339D, S371L, S373P, S375F, K417N, N440K, G446S, S477N, T478K, E484A, Q493R, G496S, Q498R, N501Y, Y505H, T547K, D614G, H655Y, N679K, P681H, N764K, D796Y, N856K, Q954H, N969K, L981F (Omicron BA.1); T19I, Δ24–26, A27S, Δ69–70, G142D, V213G, G339D, S371F, S373P, S375F, T376A, D405N, R408S, K417N, N440K, L452R, S477N, T478K, E484A, F486V, Q498R, N501Y, Y505H, D614G, H655Y, N679K, P681H, N764K, D796Y, Q954H, N969K (Omicron BA.4/BA.5). Lentiviral particles were pseudotyped with the SARS-CoV-2 spike protein from either the WT strain or the variant strains as previously described [30]. Pseudoviral particles were frozen at −70 °C for later use in neutralization assays. Titers of the particles obtained were determined based on the HEK293-hACE2 cells.

### 4.4. Pseudotyped Virus Neutralization Assay 

A pseudotyped virus neutralization assay was performed as previously described [29]. Briefly, serial diluted sera (1:4, 1:16, 1:64, 1:256, 1:1024, 1:4096) were incubated with pseudoviral particles for 1 h at room temperature, added to HEK293-hACE2 cells seeded in a 96-well plate and placed into a CO_2_ incubator for 4 days (37 °C, 5% CO_2_). Neutralization titers (ID_50_) were determined as the inverse of the serum dilution required to reduce the percentage of GFP-positive target cells by 50% compared to cells treated with pseudoviral particles without serum. ID_50_ values were calculated using non-linear regression curves fit with Sigmoidal, 5PL (GraphPad Software, San Diego, CA, USA, version 8.0.1). The baseline of neutralization titers was reported as 4. It is the maximal ID_50_ observed in the 10 pre-pandemic sera samples (the range was from 0, which means no neutralization was observed, to 4, median = 1). As a pre-pandemic control, we used serum samples cryopreserved no later than September 2019. The control and study groups were similar in terms of age and male/female ratios.

### 4.5. Antigenic Cartography

The antigenic cartography approach is based on modified multidimensional scaling [3]. The “Racmacs” package (https://acorg.github.io/Racmacs/, accessed on 15 May 2023), version 1.1.35) was used to create antigenic maps with 1000 optimizations. Antigenic maps represent a geometric interpretation of the relationships between neutralization titers of sera and viral antigens in a 2D space. One grid line on the maps (antigenic unit, AU) corresponds to a twofold dilution of sera in the virus neutralization assay.

### 4.6. Statistical Analysis

The non-parametric analogous of ANOVA for matched data (the Friedman test), with Dunn’s multiple comparisons (titers of variants compared to the WT or titers compared to each other before and after the booster vaccination), was performed using GraphPad Prism software, version 8.0.1. Data on the graphs are presented as medians and interquartile ranges (IQR). *p* values below 0.05 were considered statistically significant.

## 5. Conclusions

Our study contributes to the discussion on vaccine regimens and the methods of determining cross-reactivity of antibody repertoires. We show that the heterologous booster with Comirnaty following the primary vaccination with Sputnik V broadens the neutralization activity of sera to a greater extent than the homologous Sputnik V/Sputnik V booster. However, regardless of the nature of the booster used, the most recent of the studied variants, BA.1 and BA.4/BA.5, are still antigenically distant from the ancestral variant, which is used in both vaccines. The implications of these findings may be significant when considering a new vaccine regimen.

## Figures and Tables

**Table 1:** Participant characteristics.

Name of Subgroup		Uninfected Donors, Sputnik V Booster	Uninfected Donors, Comirnaty Booster	Convalescent Donors, Sputnik V Booster
Number of participants		39	12	7
Age	Years, median (range)	27 (18–73)	25 (19–43)	23 (19–63)
Sex	Female	19	6	3
	Male	20	6	4
Booster vaccine		Sputnik V	Comirnaty	Sputnik V
Time between prime vaccination and pre-boost serum sampling	Days, median (range)	195 (176–270)	275 (261–335)	207 (183–282)
Time between pre- and post-boost serum sampling	Days, median (range)	33 (29–39)	38 (30–46)	31 (30–36)
Anti-N IgG antibodies in pre-boost sera	Number of participants with a positive result	0/39	0/12	5/7
Anti-S IgG antibodies in pre-boost sera	Relative units (minimum—0, maximum—15.0)	6.0 (0–10.4)	7.3 (0–11.8)	12.7 (11.2–14.4)
PCR-confirmed COVID-19	Number of participants with a positive result	0/39	0/12	7/7
Time between recovery from COVID-19 and boost	Days, median (range)	-	-	78 (42–102)
Infection period		-	-	May–August 2021

## Data Availability

The data that support the findings of this study are available from the corresponding author upon reasonable request.

## Supplementary Materials

The following supporting information can be downloaded at: https://www.mdpi.com/article/10.3390/ijms241310493/s1.
