# Supplementary figures and images for: Antigenic Cartography Indicates That the Omicron BA.1 and BA.4/BA.5 Variants Remain Antigenically Distant to Ancestral SARS-CoV-2 after Sputnik V Vaccination Followed by Homologous (Sputnik V) or Heterologous (Comirnaty) Revaccination
Source: Int J Mol Sci. 2023 Jun 22;24(13):10493. doi: 10.3390/ijms241310493 (PMC10341525; doi:10.3390/ijms241310493)

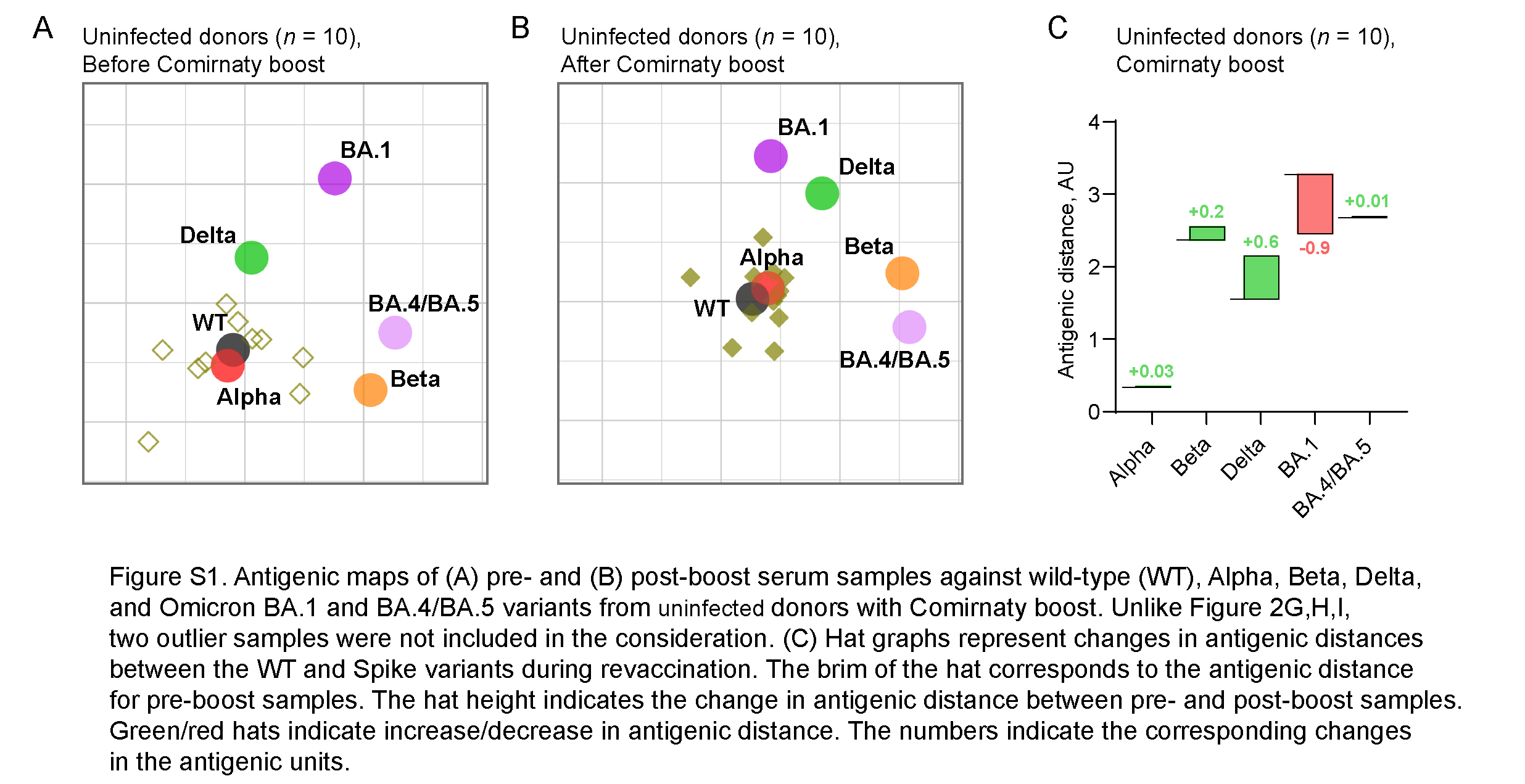

Supplement: Supplementary file 1 [file ijms-24-10493-s001.zip › ijms-2427399-supplementary.jpg]
